# Living Technology: Theoretical Perspectives on the Emergence of Sled Dogs

**DOI:** 10.3390/ani16193148

**Published:** 2026-10-08

**Authors:** Emma Vitale, Mikkel-Holger S. Sinding, Tatiana R. Feuerborn

**Affiliations:** 1 The Globe Institute, Faculty of Health and Medical Sciences, University of Copenhagen, Øster Farimagsgade 5A, 1353 Copenhagen, Denmark; mikkel.sinding@sund.ku.dk (M.-H.S.S.); tatianafeuerborn@palaeome.org (T.R.F.); 2 Qimmeq Project, Ilisimatusarfik University, Nuuk 3900, Greenland; 3 Department of Birds and Mammals, Greenland Institute of Natural Resources, Nuuk 3900, Greenland

**Keywords:** domestication, sled dogs, human–animal relations, environmental history, technology, Arctic, cultural history

## Abstract

In recent years, the intersection of the history of technology, environmental history, and ethnozoology has generated new insights into human–animal relationships, particularly in Arctic contexts where survival has depended on close interspecies collaboration. Sled dogs, which have long played a central role in Arctic lifeways, exemplify how animals function as active participants in socio-ecological systems and can be understood as forms of biological technology. Through domestication, selective breeding, and sustained practices of training and care, sled dogs developed physiological, behavioural, and genetic traits associated with endurance, thermoregulation, metabolism, and cooperative work. At the same time, humans developed harnesses, sleds, and mobility practices designed to work in tandem with canine capabilities, forming integrated transportation systems. Building on this perspective, we propose a transportation hypothesis of dog domestication. Rather than viewing transport as a late application of already domesticated dogs, we argue that mobility may have been one of the principal technological functions through which the human–dog partnership became evolutionarily and culturally stable. Under this framework, domestication is understood not only as a biological process but also as a technological one, shaped through the co-development of animals, transport systems, and human knowledge. This perspective broadens technological and ethnozoological inquiry and highlights the role of domestication and cross-cultural knowledge in Arctic human–animal relationships.

## Simple Summary

Sled dogs have been essential to life in the Arctic for centuries, helping people travel, hunt, and transport goods across challenging environments. Although they are often viewed as working animals and cultural symbols, their role as a form of technology has received less attention. This article explores how sled dogs can be understood as living technologies that developed through long-term relationships between humans and animals. We argue that transportation may have been one of the earliest and most important reasons for the development of the human dog partnership. As people moved across northern landscapes, dogs that could assist with carrying resources and pulling loads may have provided a significant advantage, creating strong incentives for continued cooperation, breeding, and training. Over many generations, these practices produced animals with physical and behavioural traits suited to endurance, mobility, and collaborative work. Together with sleds, harnesses, and travel knowledge, sled dogs formed integrated transportation systems that enabled human movement and survival in Arctic environments. By examining these relationships through the lens of technology, this article shows how domestication was not only a biological process but also a technological one, shaped by the co-development of humans, animals, and transport systems over thousands of years.

## 1. Introduction

The relationship between humans and dogs represents one of the oldest and most influential technological partnerships in human history. Since the Late Pleistocene, dogs have accompanied people as hunters, companions, guards, and transport animals, enabling humans to occupy environments that would otherwise have been difficult to exploit [1,2,3,4]. Nowhere is this relationship more evident than in the Arctic, where the collaboration between humans and dogs has shaped patterns of mobility, subsistence, exploration, and cultural development for millennia.

Mobility has long been a defining challenge of Arctic life. Vast distances, highly seasonal resources, and extreme environmental conditions demanded efficient systems for transporting people, food, equipment, and materials across snow and sea ice. Archaeological discoveries from the Zhokhov site in the New Siberian Islands, including sled components and dog remains dating to approximately 9000 years ago, demonstrate that dogs were already functioning as draft animals during the Early Holocene [5,6]. Since then, dog sledding has become one of the most enduring technological systems of the circumpolar North. Dogs facilitated hunting, trade, communication, and travel across otherwise inaccessible landscapes for the ancestors of indigenous populations or Arctic North America, the Inuit, Yu’pik, and Aleuts. They later enabled some of the most significant Arctic and Antarctic expeditions, including Knud Rasmussen’s Fifth Thule Expedition, the Denmark Expedition led by Ludvig Mylius-Erichsen, and Roald Amundsen’s successful journey to the South Pole [7,8].

Despite their historical importance, sled dog populations have declined substantially in recent decades. In Greenland alone, the registered sled dog population decreased from approximately 25,000 animals in 2002 to around 15,000 by 2016, with numbers continuing to decline due to climate change, diminishing sea ice, socioeconomic transformation, and reduced reliance on subsistence hunting [9]. This decline threatens not only a distinctive dog population but also one of the world’s oldest continuously operating human–animal technological systems. Beyond their archaeological and cultural significance, these findings have important conservation and welfare implications. Protecting sled dog health, genetic diversity, and working conditions is essential for sustaining both animal welfare and the cultural heritage embedded in Arctic human–dog relationships. Continued population declines may therefore have consequences that extend beyond the dogs themselves to the preservation of Indigenous knowledge, traditional husbandry practices, and the long-term viability of one of the world’s oldest human–animal working partnerships [9]. However, there are ongoing local initiatives that aim to protect the endangered Greenlandic sled dog and its cultural heritage through year-round sustainable tourism, hands-on visitor education, and local breeding traditions.

Traditionally, technology has been associated with mechanical inventions and industrial development. Such perspectives have tended to separate technology from nature, reinforcing the assumption that technologies are necessarily manufactured, inert objects [10,11]. Yet throughout human history, many of the most transformative technologies have been living. Domesticated plants and animals have fundamentally altered human societies through agriculture, transport, communication, and labour. The domestication and selective breeding of animals therefore constitute technological practices in their own right, producing living systems whose biological characteristics have been shaped through sustained human intervention [12,13,14].

Sled dogs exemplify this relationship between biology and technology. A dog team is more than a collection of animals pulling a sled; it is an integrated technological system in which humans, dogs, harnesses, sleds, and the environment operate together. The effectiveness of this system depends upon continual communication, cooperation, and mutual adaptation. Commands, body position, terrain, weather, and the responses of individual dogs all contribute to a dynamic process in which technology is enacted through movement rather than simply embodied in an artefact. The musher and the dogs function as a coordinated technological unit, blurring conventional distinctions between operator and tool, nature and technology [15,16].

Viewing sled dogs as living technologies also invites a reconsideration of domestication itself. Existing models of dog domestication have primarily emphasized commensal scavenging, hunting partnerships, or social companionship as the principal selective forces driving the emergence of domestic dogs [17,18]. While these explanations have substantially advanced our understanding of domestication, they pay comparatively little attention to mobility as a technological challenge. If transportation became essential to human survival and expansion in northern environments, it is plausible that the capacity of early dogs to assist with moving people and resources represented one of the earliest sustained selective pressures reinforcing domestication.

Building upon this perspective, we propose a transportation hypothesis of dog domestication. Rather than viewing transport as a relatively late application of already domesticated dogs, we argue that transportation may itself have constituted one of the principal technological functions through which the human–dog partnership became evolutionarily and culturally stable. Under this hypothesis, domestication is understood not solely as a biological or behavioural process, but as a technological one in which selective breeding, training, and increasingly sophisticated transport systems co-evolved over thousands of years.

This article contributes to the history of technology by reframing sled dogs as living technologies and by positioning transportation as a potential driver of dog domestication. Through this approach, we argue that domestication should be understood as both an evolutionary and a technological process, one in which humans and dogs co-created a living system that transformed mobility, enabled Arctic adaptation, and continues to embody a unique form of intangible cultural heritage.

## 2. Background: The Versatile Role of Dogs in Arctic Societies

Dogs have played an essential and diverse role in many northern communities, especially in Arctic societies [19]. Sled dogs have supported humans in hunting and herding activities and have been widely used for transportation [1,20,21,22]. They also served as food sources, ceremonial offerings, and appear in Inuit cosmology and mythology. Dogs had multiple functions not only during their lives but also after death [21,23]. Archaeological excavations in North America and Siberia have uncovered dog bones with cut marks, indicating they were butchered [24,25]. For example, at the Ust’-Polui site in Siberia, many dog bones show signs of slaughter [25]. Dog remains were also used as raw materials, such as for clothing [26]. Sled dogs have a thick, three-layered coat that allowed them to survive Arctic conditions, a material that humans used to protect themselves from the cold [26]. We suggest that the technological advance provided by dogs in the form of transportation, through pack, sled, travois, and other mediums, shaped the early dogs during the domestication process in Eurasia.

Sled dogs constitute a distinctive form of living technology, not merely as working animals but as biological actors possessing specific characteristics that makes them essential within Arctic transport mobility systems. Across the circumpolar Arctic, sled dogs have collaborated with humans for more than 9000 years, underscoring both their technological significance and their cultural value [6,27]. Arctic sled dog types, including the Siberian Husky, Alaskan Malamute, Canadian Inuit Dog, and the Greenlandic sled dog (Qimmeq), share close common ancestors but have developed specialized adaptations to the Arctic and sledding performance [27,28]. The Greenlandic sled dog is of particular interest, as this population has retained its functional role as a sled dog in Greenland for nearly a millennium, without the extensive intervention or transformation into companion animals that other Arctic breeds have undergone [28]. In fact the migrations of humans can be traced through the ancestry of dogs in the Arctic, underscoring their role within the suite of tools of arctic societies [1,27,28,29]. Genetics have established that the genetic ancestry that defines sled dogs genetically was present in Siberia already 9700 years ago as evidenced by the genome of a dog originating from Khatystyr cave in Southeast Siberia [30]. It has also been suggested that thermal reception, an Arctic key evolutionary trait [31,32], was already under selection in the dogs, as well as hypoxia adaptation, likely related to the extreme athletic performance of the dogs during sledding as seen in the Zhokhov dog genome [27]. Together this suggests that by the early Holocene the sled dog lineage that continues today was already specialised for sledding and Arctic conditions. Interestingly, the modern sled dogs show genetic adaptation to a diet rich in fatty acids [27], likely a result of humans feeding them marine mammal blubber over generations. However, these mechanisms are not evolved in the ancient Zhokhov dog [27], indicating that across time sled dogs have continued to evolve, and as living beings will continue to do so. Greenland is unique in that the Greenland Sled Dog continues to live and work within the Indigenous sledding culture in which it evolved, preserving a human–dog relationship that has persisted since the arrival of the Thule Inuit more than 800 years ago [28]. Amazingly, the adaptation to cope with a diet rich in fatty acids is an ability co-evolved in polar bears [33] and Arctic humans as the Inuit [34,35]. Being a biological example of convergent evolution and further connecting sled dogs and Arctic cultures, they have followed each other for so long that they have evolved in the same direction.

The evolution, ancestry, physiology and behaviour of the sled dog reflect its life across millennia and the living dogs embody all this legacy. These dogs are robust, possessing dense coats, strength, and endurance, and they exhibit social and communicative capacities that enable them to operate within complex teams. Sled dogs acquire skills through practice and socialization within the pack, where hierarchy and cooperation are essential for effective sled travel [22]. These characteristics are not static but develop through ongoing interactions with humans, the landscape, and technological objects such as sleds and harnesses [16].

There is strong archaeological evidence for the use of sled dogs during the Thule period (1200-1850 CE) across the North American Arctic [36]. These dogs originally came from Siberia and spread through the Bering Strait into central and eastern Arctic regions [1]. This movement aligns with the spread of related technologies found in archaeological records [36]. Sled dogs enabled long-distance travel, connected communities, and supported the use of marine resources. European expeditions in the Arctic relied heavily on Inuit knowledge and their use of dogs for transport. In this context, dogs acted as resilient partners and as a bridge between traditional ways of life and new challenges, continuing to support mobility. Throughout historical transitions, the presence or absence of sled dogs shows that they have been active participants in the adaptation of Arctic life ways. By enhancing human capacity to respond to environmental, spatial, and cultural challenges, dogs have played a key role in innovation and change in the Arctic.

Activities such as sledding and hunting integrate dogs’ physical capacities with human planning, tool use, and social organization, including speed, climate resistance, and tracking ability. Together, they form a technology that neither species could achieve alone. This cooperation has enabled Arctic peoples to understand and interact with their environment in new and creative ways. Sled dogs have thus contributed to human development and helped communities adapt to change. They have enabled movement across tundra, ice, and sea, and supported adaptation and resilience in a landscape shaped by constant transformation [16]. The many roles of sled dogs, from transport and hunting to protection, demonstrate their remarkable versatility and adaptability. Their long-standing relationship with humans reflects a mutual domestication process or co-domestication [22,37,38]. This form of coexistence offers insight into the complex and dynamic relationships between humans and animals throughout history and deepens our understanding of how these relationships have shaped both cultural practices and technological systems.

## 3. Domestication and Living Technologies

The timing, geographical, and cultural context of dog domestication remain debated, although archaeological and genomic evidence increasingly indicates that the process began during the Late Pleistocene in Eurasia [4]. This precedes the appearance of clearly recognisable archaeological dog remains in the early Holocene including the widely accepted Bonn Oberkassel dog dated to approximately 14,000 years ago [39]. Recent ancient genomic studies have demonstrated that dogs were already genetically distinct from wolves by at least approximately 15,800 years ago [2,4] and genomic divergence estimates have placed the separation of the dog lineage from wolves considerably earlier, potentially during the period between approximately 20,000 and 40,000 years ago [40,41,42]. The geographic location of this process remains unresolved, although it is widely accepted that it occurred in Eurasia. Rather than supporting a single, definitively established centre of domestication, the available evidence is therefore consistent with a complex process occurring within Late Pleistocene hunter–gatherer societies, with suggestions of dual domestication [43], early diversification [44], early specialisation [27], and widespread movements of dogs [4,26,30]. Importantly, the substantial chronological gap between the inferred origins of the domestic dog and the evidence for specialised working dogs presents a challenge for determining the mechanisms and motivations that led to the dog lineage.

When wolves were first domesticated, humans had no prior experience with domestication, making it unlikely that they had a clear plan or goal, such as turning wolves into herding dogs. One of the key components in the mystery of the origin of domestic dogs is what could possibly make an efficient reproductive barrier between early dogs and wolves, allowing the common ancestry of all dogs to drift sufficiently away from wolves becoming the clear lineage that dogs represent. Potentially sledding ability could be such a barrier. The authors would like to underline that this idea remain an explicitly limited hypothesis, as no evidence from the actual context of dog domestication can be provided. The transportation hypothesis relies on observations from traditional sled dog breeding, which corroborates that sledding performance is a barrier to admixture with wolves, entertaining the idea of whether similar mechanisms have played a role as a barrier in the past. Traditional knowledge from Inughuaq hunters and mushers in Avanersuaq (North Greenland) with experience in working with wolf–sled dog hybrids explain how the first generation of wolf dog offspring are unable to pull a sled efficiently, due to their body shape, and that several generations of out breeding with dog ancestry is needed to have a “sled dog” with part wolf ancestry [45]. The low utility of hybrids to pull sleds due to their legs being too long is further supported as mentioned in historical anecdotes. Specifically in the context of Greenland sled dogs, where occurrence of wolf hybridization is documented [45,46], it is noteworthy that no Arctic wolf ancestry have been detected in over 100 dogs that have been genome-sequenced and tested [27,28]. Pointing out that, while wolf–dog hybrids do occur, they have not contributed significantly to the overall gene pool of Greenland sled dogs, with the poor sledding performance of hybrids being a possible contributing factor. If early dogs obtained or had adaptations favourable in sledding, that would put a selection pressure on wolf hybrids. In this way sledding could be a bottleneck of reproductive isolation from wolves in the common ancestry of all dogs, a tradition maintained in modern sled dogs, but lost in other dogs as their ancestors diversified into other ways of life across human cultures. Taming is often seen as the first step in the domestication process, but coincidences likely played a major role. Taming changes behaviour, while domestication changes both behaviour and genetics. Domestication represents a continuous process that relies upon the sustained, multigenerational influence over breeding strategies to produce a lineage shaped by the partner organism [17,18]. A likely scenario is that domestication happened gradually through many small, mostly unintentional steps. These changes were driven by immediate, practical needs rather than long-term breeding strategies. One possible starting point was wolves living near human settlements, feeding on waste, like bones and food scraps. Humans may have tolerated, or even appreciated, these wolves, as they helped dispose of waste and could alert people to danger from wild animals or strangers. Genetic research has supported this with the identification of genes under selection in dogs that are associated with starch digestion [47]; however, these adaptations may have followed the onset of the Neolithic when cereals were domesticated [48], which is supported by the absence of these adaptations in Arctic dogs [27]. Wolves that were less fearful of humans had better access to food and were more likely to survive and reproduce. Over time, this led to natural selection favouring less fearful, more human-friendly wolves. Eventually, some of these wolves may have entered human camps and been treated as semi-domesticated animals, providing companionship or serving as food sources. When deciding which animals to kill, humans likely removed the most aggressive ones first. This would have further encouraged willing behaviour in the population. Over generations, these ‘proto-dogs’ began to look and behave very differently from wild wolves and were eventually recognized as the dog. This long-term process led to significant behavioural and physical changes, creating a distinct population.

Although dogs are considered the first domesticated animals, domestication should be viewed as an ongoing process. The relationship between humans and dogs is best described as co-domestication, where both species adapt to each other [22]. To understand this process, it is important to move away from a human-centred view and instead highlight the role of animals in history [48]. In this multispecies perspective, objects often act as mediators between humans and animals. For example, McNiven [15] describes the canoe as a tool that mediates the hunting relationship between humans and marine animals. Other technologies, such as tools or navigation skills, are also key to how northern peoples interact with working animals [37]. In northern societies, domestication often takes place in shared landscapes where humans and animals live together. The interaction between animals, landscapes, tools, and people is essential to understanding domestication. The landscape itself connects species and shapes their relationships [49]. In this ecological system, cooperation between humans and dogs is grounded in the environment. The relationship between animals and technology is mutual. Technologies are not just tools for managing animals; animals and animistic beliefs also help us understand technology.

Thus, as relationships between humans and wolves became more established, additional opportunities for cooperation may have emerged. While hunting is often proposed as one of the earliest functional roles of dogs, transportation represents another possible context for the development of human–dog cooperation. Archaeological evidence for early transport technologies provides tentative support for considering mobility in this context, although it does not by itself demonstrate that dogs were used for traction [6,50]. We propose that use in transportation was an early driving force that propelled the adoption of proto-dogs or wolves into domestic environments that lead to the distinct animals we now know as dogs. Archaeological evidence indicates that sledding technologies were present in the Late Pleistocene; however, even before the invention of formal sled technology, wolves or early dogs may have assisted people by carrying light loads or dragging simple travois, reducing the energetic costs of transporting resources across landscapes that would be challenging to identify in the archaeological record. In highly seasonal northern environments, where mobility was essential for following game and moving camps, even modest assistance with transport would have provided a considerable advantage. Individuals that were more tolerant of human direction, more willing to work cooperatively, and physically capable of pulling loads would have been preferentially retained and reproduced, strengthening selection for behavioural traits associated with domestication. Although direct archaeological evidence for the earliest use of dogs in transport remains scarce, the antiquity and widespread distribution of sled dogs across the Arctic suggest that transportation may have been one of the earliest specialised roles that reinforced and accelerated the domestication process, rather than simply representing a later cultural innovation. Furthermore, the environmental condition present at the time of the divergence of the dog and grey wolf lineages was colder than the present day; as a result conditions supporting sledding would have been more widespread across Eurasia as suggested by palaeoenvironmental reconstructions [51,52,53]. For highly mobile hunter–gatherers, particularly those inhabiting seasonal and resource-variable environments, efficient movement was fundamental to survival. Even before the emergence of sophisticated sleds, early dogs may have assisted by carrying loads or dragging simple transport devices, increasing hunting efficiency, facilitating residential mobility, and expanding the distances over which people could transport food, raw materials, and equipment. Individuals that were more cooperative, tractable, and responsive to human direction would consequently have been preferentially retained and bred. Transportation therefore acted not simply as a use of dogs, but as a technological niche that favoured behavioural and physiological traits associated with domestication itself. In this framework, mobility became a driver of co-evolution [51].

### Domestication Through Material Culture

**Figure 1:**
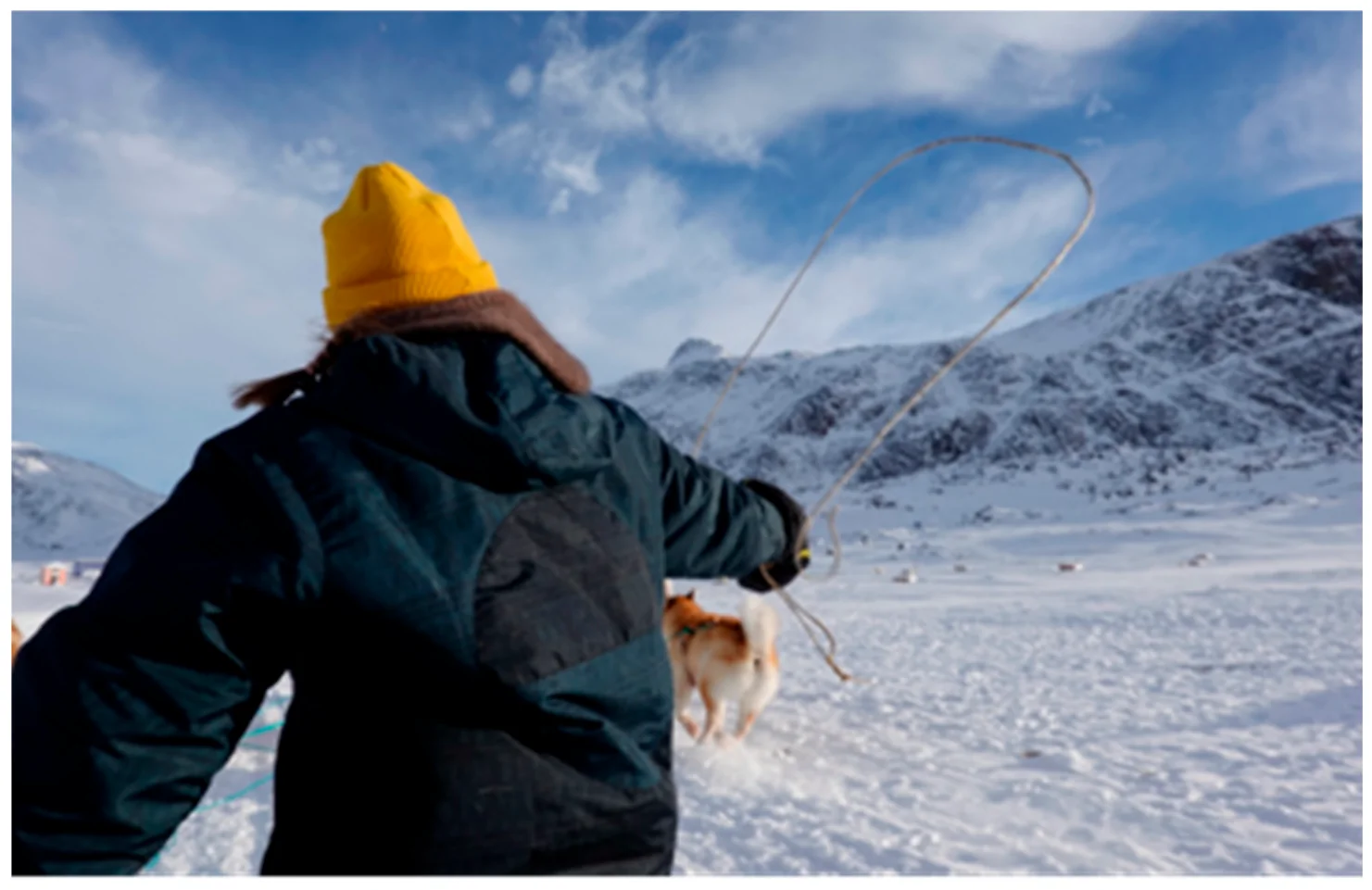
A musher, Julia Lings of The Greenland Dog Adventure and Preservation program, in Sisimiut, Greenland, employing a dog whip as a communicative tool to direct the sled dog team. Photo by Emma Vitale.

In Arctic human–animal relationships, material culture and environmentally situated practices are often central to the development of shared systems [16]. The ongoing domestication of dogs, specifically sled dogs, involves skill learning by both humans and animals. This mutual learning is essential to the cooperation between sled mushers and their dogs. Acquiring a skill, as required by technology, is a key part of the domestication process. It involves repeated actions and develops over time. Dogs learn to understand verbal and non-verbal communication, procedures, and gestures, while humans learn how to guide and respond to the dogs. Communication between the musher and the dogs often involves harnesses, whips and buckles (Figure 1), not just voice or body language. These tools have been continuously refined for this purpose. Therefore, technical learning must be mutual and based on shared experience and cooperation, not just imposed on the animal [38].

Objects like dog whips and harnesses can be seen as tools through which humans can communicate their intention to the dogs (Figure 1). When considering how dogs and humans interact physically, harnesses in particular bring the two species into contact and signal the next step, such as preparing the dog for sledding. These tools also create a framework for domestication and act as social infrastructures. If seen only as instruments of human control, they fail to support communication in a shared landscape of coexistence. Becoming a working animal involves many factors, including the physical environment, the nature of the landscape, and the practical knowledge held by both experienced mushers and dogs.

## 4. Animals as Technological Agents

To understand sled dogs as part of the history of technology, we must consider what kind of system they are part of. In the Arctic, the sled dog system can be seen as a network that includes dogs, humans, sleds, harnesses, feeding strategies, and breeding practices. Together, these elements form an integrated system that enables transportation and survival in extreme environments. Approaches like Actor-Network Theory (ANT) [54,55] and Human–Animal Studies (HAS) offer useful frameworks for analysing such systems. These perspectives discuss humans, animals, and tools as interconnected actors that influence and shape one another.

Sled dogs are not passive tools in a technological system; instead, they are co-actors with a degree of autonomy and unpredictability. This view aligns with recent work in environmental history and multispecies studies, which emphasize the dynamic and reciprocal nature of human–animal relationships [56]. The idea that animals can be part of technological systems has gained attention in the history of technology and multispecies research. These fields challenge the traditional view of technology as only non-living, human-made objects. Instead, they propose a broader definition that includes living beings, such as domesticated, trained, or bred animals, as technological components [10,13]. Some scholars argue that animals like sled dogs can be seen as biotechnological agents, functioning within infrastructures and production systems alongside machines and tools [57]. Others, especially in posthumanism and multispecies studies, caution against reducing animals to mere tools. They emphasize the agency of animals and argue that technological relationships should be understood as mutual and context-dependent [56,58]. Using ANT and HAS allows for a more nuanced analysis of sled dogs as technology. This includes their biological abilities, social roles, and interactions with material tools. This perspective expands our understanding of technological systems by including living beings as active participants, challenging the traditional boundaries between technology, nature, and culture.

Previous studies have explored animals, especially horses, as both workforce and living machines [10,57,59]. This perspective connects animal technologies to broader discussions in environmental and technological history. Technologies shape who we are, and animals have often been studied in three main technical categories: agriculture and food production; energy and transport; and animal-based scientific studies (e.g., biotechnology, ecology, evolutionary biology, and zoology). In Horses at Work [10], Greene shows how studying animal technologies can enrich the field of technology history by linking them to cultural, economic, and military contexts. Just as horses, dogs can be analysed as part of a technological system, whose historical role helped shape human societies. Cultural history helps us understand societies consisting of both humans and animals, living and working together under shared, but unequal, social norms. In the history of technology, which is a dynamic and evolving field, it is important to recognize that animals can be powerful, material, and human-shaped, like machines, but also living, sensing, and conscious beings. This challenges the clear division between human and non-human actors [60].

Dogs are not just biological beings, they are the result of millennia of co-evolution and technological interaction, optimized for specific roles in environments. As humans play an increasingly active role in ecological and evolutionary processes, it becomes more important to recognize living beings as integral parts of technological systems, both as material resources and as co-creators of human experience and culture [61]. Sled dogs are not just working animals; they represent a living cultural heritage and evidence to the long-standing and complex relationship between humans and animals in the Arctic [62]. To understand the continued relevance of sled dogs in technological and cultural systems, we must recognize their role not only as animals but as carriers of knowledge, tradition, and identity. Sled dogs embody intangible cultural heritage, a body of knowledge, stories, sounds, and movements that cannot be learned from books but only through lived experience. This knowledge is passed down through generations, including through the dogs themselves [56,58]. Recognizing sled dogs as intangible heritage is essential to understanding their full significance [62]. It is not only about preserving a tradition, but about maintaining a living relationship between humans, animals, and the landscape, one that includes technical skills, emotional bonds, and legacy.

### 4.1. Dogs as Part of Technological Systems

The discussion regarding whether animals can be considered technology likely comes from the influence of the Industrial Revolution. This period emphasized machines replacing nature, such as horses being replaced by automobiles, leading to the belief that only non-living machines count as technology. However, throughout history, humans have used many tools, some mechanical, others not, all of which are forms of technology. These tools often come from nature, which humans have optimised for their needs [11,14]. Although many technologies consist of non-living materials, some tools are living organisms. Organisms become tools when they are used to meet human needs or purposes. Most domesticated plants and animals have been transformed and can be considered as human-made objects [13]. Terms like “biotechnology” and “genetic engineering” show how biological and artificial systems combine to create new tools and technology. For around 10,000 years, humans have turned wild species into domesticated ones to support farming. All established societies rely on biotechnology, recognizing this long history helps us understand modern genetic engineering [13]. One way to consider animals as technology is to perceive them as products, used for transport, food, or companionship. Breeding has also aimed to standardize these products; dog breeders are skilled in this, and even people unfamiliar with dogs can often recognize different breeds. The idea of a “pedigree” has become a symbol of quality in dog breeding [63].

Animals have also performed as a workforce. Sometimes they replace or support human workers, like herding dogs helping shepherds, at a lower cost than human labour [64]. In other cases, animals do tasks humans cannot, such as detecting bombs or drugs with their strong sense of smell. Animals have also been used instead of machines [61].

### 4.2. Sled Dogs as Part of Arctic Technological Systems

**Figure 2:**
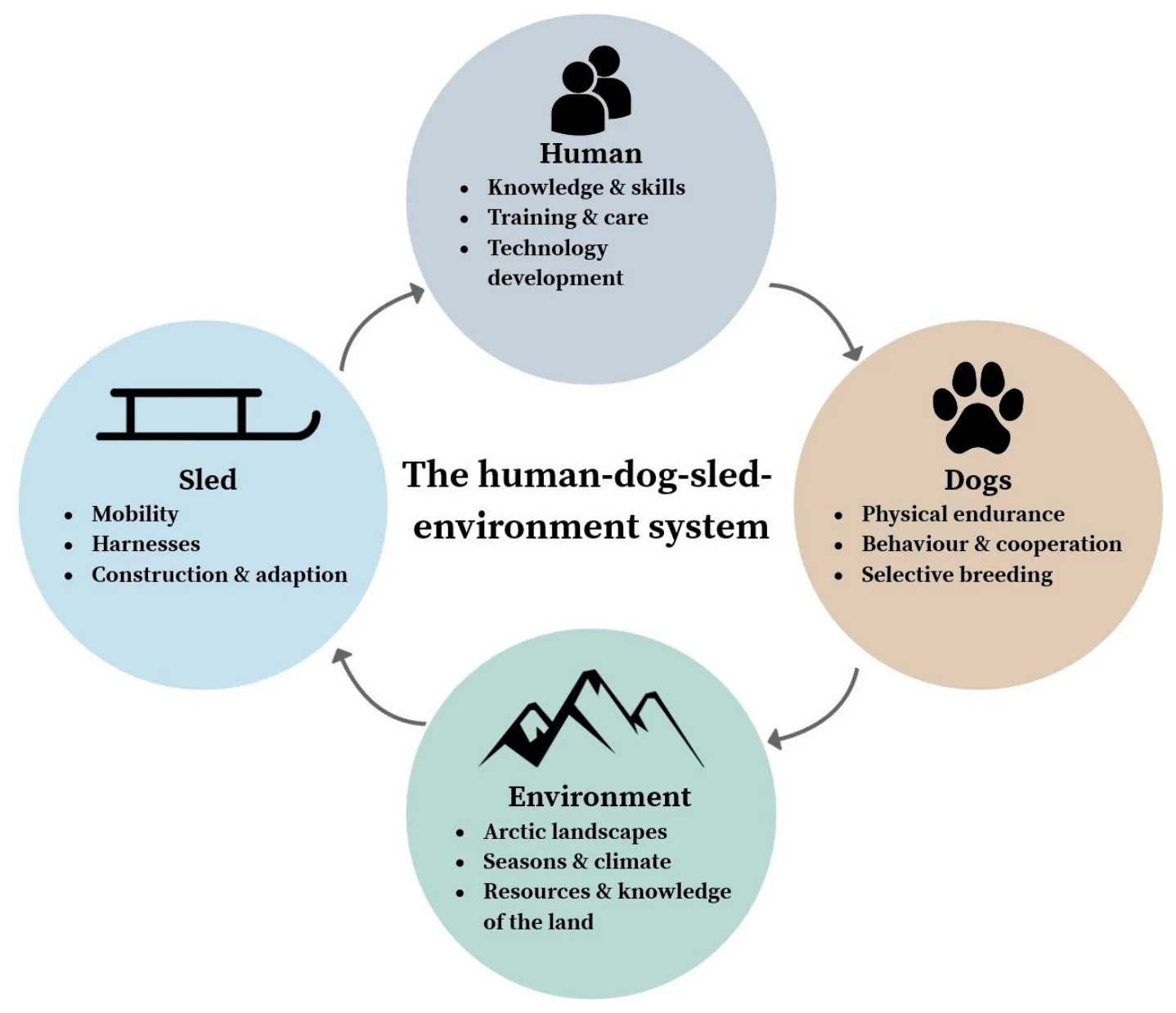
Schematic representation of the proposed human–dog–sled–environment system underlying the transportation hypothesis of dog domestication. Arctic environmental conditions created strong mobility demands that were met through the co-development of human travel practices, sled and harness technologies, and sled dog physiological and behavioural traits. Together, these elements formed an integrated transportation system that supported survival, mobility, and long-term co-evolutionary relationships between humans and dogs.

For centuries, dogs have been used for transport in the North because they are well adapted to the environment. Arctic communities have also tamed reindeer for milk, pulling loads, and carrying goods [65]. Studies of horses as living machines help us understand sled dogs as technology in Arctic societies [10,57]. Like horses, sled dogs were essential for transport, but they also had social and cultural roles. Their use involved breeding, equipment, and human interaction, showing that animals are not just tools but active parts of technological systems. Research on human–animal relationships and animal technology shows how sled dogs were more than just transport; they were part of a system shaped by social, economic, and environmental factors (Figure 2) [60]. As new technologies entered the Arctic, it became important to understand how they changed human–animal–technology relationships. The shift from dog sleds to snowmobiles shows that modernization is not just about efficiency; it also affects culture and adaptation. Since the mid-20th century, many Inuit and Arctic communities have adopted snowmobiles. Although snowmobiles are fast and can carry heavy loads, they have not fully replaced sled dogs. In fact, across the Arctic both are now used together. Many traditional hunters still see sleds as more reliable and durable [66].

In Greenland, recent studies show that switching from sled dogs to snowmobiles is not just about technology; it also involves identity, tradition, and climate change [16,67]. In Kulusuk, East Greenland, people say snowmobiles changed how they move around, but sled dogs still matter for hunting, tourism, and cultural heritage [67]. Similar trends are seen in Alaska, where motorized transport has deeply affected both the environment and community life. According to Francis [68], this shift has changed the social and economic system, risking the loss of traditional knowledge. In the Canadian Arctic, this has been called the “snowmobile revolution,” where snowmobiles largely replaced sled dogs in the 1960s. This change was driven by practical needs and government modernization efforts, but it also sparked debates about losing cultural continuity and sustainability [64,69].

Due to climate change, changing ways of living, and geopolitical shifts, traditional sled dog practices and their cultural heritage are increasingly at risk. Understanding the long-term relationship between humans and dogs is important not only for learning about past survival strategies but also for preserving the genetic and cultural heritage of these animals [70]. These practices formed complete technological systems, yet sled dogs have often been ignored in studies of 19th- and 20th-century technology, despite their key role in Arctic life. Separating sled dogs from technology is in contrast to how society perceived them within the Arctic. Fascinated by mechanics, people often used technological metaphors to describe and understand sled dogs as sources of power and to find ways to improve their endurance, strength, and efficiency [66]. What is considered technology changes over time. To understand Arctic technology history, we must recognize how past societies understood and used technology, not just as machines and tools, but as living actors in a dynamic relation between humans, animals, and landscape (Figure 2).

## 5. Conclusions

Technology is not limited to mechanical devices, and many technologies were developed before machines were invented. The domestication and use of animals have played a central role in the development of technological knowledge and practice [12]. Sled dogs are an example of this. Over thousands of years, they have been selectively bred and trained to perform specific tasks in Arctic environments, making them living technologies or biological systems adapted to human needs in the Arctic.

Building on this perspective, we propose the Transportation Hypothesis as a framework for considering whether mobility and transport may have contributed to the development and specialization of the human–dog relationship. Existing models of dog domestication have primarily emphasized commensal scavenging, hunting partnerships, or social companionship as important contexts for the emergence of this relationship. While these explanations are not mutually exclusive, they may not fully account for the technological significance of mobility. The Transportation Hypothesis therefore proposes that, once early wolves became sufficiently tolerant of humans, their capacity to reduce the energetic costs of moving people, camps, and resources may have provided an additional context in which human investment in dogs became advantageous. Importantly, this hypothesis does not establish transportation as the cause or initiating mechanism of dog domestication. Rather, it identifies transportation as a potential technological dimension of the longer process through which human–dog relationships developed and became increasingly specialized.

The primary challenge in the investigation of the origin of the dog is defining the moment when a dog is no longer a wolf and has instead become differentiated enough to be called a ‘dog’. This presents complications in not only determining when and where dogs would have originated but also in determining why they came to be in the first place. As a result, the precise moment will likely never be pinpointed; however, researchers can continue to seek evidence for where the earliest distinct individuals appear and what cultural context they are derived from. Locating evidence for use of dogs as aids in hunting, guarding, and companions before written records will be limited to indirect evidence such as pictorial representations. Future genetic research exploring adaptations in early dogs may uncover evidence for selective breeding for culturally valuable traits outside of those indicating the selection in sled dogs and may provide further insight into alternative explanations for the roles of early dogs.

This perspective reframes domestication as a technological process rather than solely a biological or behavioural one. Selective breeding, training, and the development of transport technologies, including harnesses and sleds, can be understood as components of a longer technological relationship between humans and dogs. The sled should therefore not be regarded as the beginning of transport technology, but as a later development within a broader history in which humans and dogs adapted to one another through shared labour and movement across landscapes. The archaeological and genomic evidence currently available does not establish that transportation initiated dog domestication, but it provides a basis for considering mobility as one possible dimension of the technological relationship between humans and dogs.

Recognising sled dogs as living technologies also highlights their significance as intangible cultural heritage. Their value lies not only in their genetics or working ability but in the accumulated body of ecological knowledge, technical skills, oral traditions, and social practices transmitted through generations of people and dogs together. This relationship is best understood as one of co-domestication, in which humans and dogs have mutually shaped one another biologically, culturally, and technologically. Conserving sled dogs therefore means preserving not only a distinctive animal population but one of humanity’s oldest continuously functioning technological systems and a living partnership through which movement, survival, and culture have co-evolved for millennia.

## Data Availability

No new data were created or analyzed in this study. Data sharing is therefore not applicable.
